# Supplementary material for: CT-Angiography-Based Outcome Prediction on Diabetic Foot Ulcer Patients: A Statistical Learning Approach
Source: Diagnostics (Basel). 2022 Apr 25;12(5):1076. doi: 10.3390/diagnostics12051076 (PMC9140120; doi:10.3390/diagnostics12051076)
Supplement: Supplementary file 1 [file diagnostics-12-01076-s001.zip › diagnostics-1656813-supplementary.pdf]

CT scanning parameters and contrast material injection protocol

CT Scanning Parameters

The multiple-detector CT (MDCT) scanners involved in this study can be summarized into three types. The detailed scanning parameters are listed in Table S1.

Table S1: Scanning Parameters for Lower extremity CTA

Equipment	Rotation Time (sec)	Detector-row Width (mm)	Pitch	Table Speed(mm/sec)	Scan Time (sec)
16-MDCT (Siemens)	0.5	0.75	1.5	18	40
64-MDCT (Siemens)	0.5	0.6	0.85	30	40-42
64-MDCT (GE)	0.5	1.25	0.516	41.2	29-31

NOTE: MDCT: multiple-detector CT

Contrast Material Injection Protocol

A total volume of 120mL of nonionic contrast material (iohexol [Omnipaque 300 mg/mL, Daiichi-Sankyo]; Iodixanol [Visipaque 320 mg/mL, Amersham Health]; Iopromide [Ultravist 300 mg/mL, Schering]) was injected through a 20-gauge catheter into the antecubital vein with a power injector. The detailed contrast material injection protocols are shown in table 2.

Table S2: Contrast Material Injection Protocol for Lower extremity CTA

Equipment	Injection Duration (sec)	Scanning delay (sec)	Injection flow rates (mL/sec)
16-MDCT (Siemens)	35	10	4
64-MDCT (Siemens)	35	10	3-4
64-MDCT (GE)	35	9-11	3

NOTE: MDCT: multiple-detector CT
